# Design and evaluation of mobile application for adolescents’ self-care: protocol for a randomized controlled field trial

**DOI:** 10.1186/s13063-024-08064-2

**Published:** 2024-03-28

**Authors:** Razieh Rezaee, Mohtasham Ghaffari, Reza Rabiei, Amir Kavousi, Sakineh Rakhshanderou

**Affiliations:** 1 Health Education and Health Promotion, School of Public Health and Safety, Shahid Beheshti University of Medical Sciences, Tehran, Iran; 2 Department of Health Information Technology and Management, School of Allied Medical Sciences, Shahid Beheshti University of Medical Sciences, Tehran, Iran; 3 Workplace Health Promotion Research Center and Department of Epidemiology, School of Public Health and Safety, Shahid Beheshti University of Medical Sciences, Tehran, Iran

**Keywords:** Mobile health, Mobile application, Self-care, Adolescents

## Abstract

**Background:**

Adolescence is a critical stage for the development of self-care. Many adolescents use mobile apps to monitor and improve their health. Health information technology plays a significant role in the field of self-care. This article provides a protocol for a study to design and evaluate mobile applications for adolescent self-care.

**Methods:**

The present research is a combination of applied development study, methodological, and intervention experimental. This study will be done in three stages: The first stage is the design and evaluation of a mobile application for adolescent self-care. The second stage is, designing and evaluating the psychometric properties of the “Questionnaire for Measuring Adolescent Self-Care Performance.” This questionnaire will be used before and after using the application in the third stage. The third stage is determining the effectiveness of self-care interventions based on mobile applications among adolescents. The target group will be adolescents aged 10–19 from the schools of Amol City. In the first stage, the opinions of 30 people adolescents, parents, and experts will be used. In the second stage, the number of samples will be 10 times the number of items in the questionnaire. In the third stage, 50 people will be in the intervention group and 50 people will be in the control group. Descriptive statistics will be used for data analysis. Between-group and intra-group comparisons will be calculated about quantitative variables, independent t-test and paired *t*-test, and analysis of variance. The chi-square test and Fisher’s exact test will be used in SPSS 16 software to test the homogeneity of qualitative variables between the two groups.

**Discussion:**

In the first stage, based on the opinions received from the target group, a user-centered educational application for self-care of adolescents will be designed. In the second stage, after determining the validity and reliability, a questionnaire will be designed to measure the self-care performance of adolescents. In the third stage, using an intervention study for 3 months, the effectiveness of the training will be determined through the designed application. Our findings are scheduled for a full analysis, with expectations that analyses will be completed by September 2023.

## Introduction

Enjoying health and well-being is considered one of the basic human needs and rights. Self-care—the first step to health—means taking care of yourself [1]. The concept of self-care was first introduced in 1959 by Orem as the “Nursing care deficit theory of self” [2]. Self-care is a conscious, learned, and purposeful practice. In it, each person uses his acquired abilities and skills in such a way that he can take care of himself personally and independently and help maintain and improve his health [2, 3].

Self-care is known to be one of the health-promoting behaviors of all ages that increases the level of health and quality of life. Health in adolescence guarantees health in adulthood and the health of future generations of any society. One of the best approaches in the field of adolescent health is to pay attention to the concept of self-care and its principles, about the physical and mental development of adolescents [4]. Therefore, the need for education and adoption of self-care behaviors is of particular importance for adolescents. In addition, raising the level of awareness of children and adolescents about the harmful factors that exist around them and teaching them how to interact properly with pathogens can play an important role in vaccinating them against such injuries [3, 5].

Adolescence is the phase of life between childhood and adulthood, from ages 10 to 19. It is a unique stage of human development and an important time for laying the foundations of good health. Adolescents experience rapid physical, cognitive, and psychosocial growth. This affects how they feel, think, make decisions, and interact with the world around them. Despite being thought of as a healthy stage of life, there is significant death, illness, and injury in the adolescent years. Much of this is preventable or treatable. During this phase, adolescents establish patterns of behavior—for instance, related to diet, physical activity, substance use, and sexual activity—that can protect their health and the health of others around them, or put their health at risk now and in the future. To grow and develop in good health, adolescents need information, including age-appropriate comprehensive sexuality education; opportunities to develop life skills; health services that are acceptable, equitable, appropriate, and effective; and safe and supportive environments. They also need opportunities to meaningfully participate in the design and delivery of interventions to improve and maintain their health. Expanding such opportunities is key to responding to adolescents’ specific needs and rights https://www.who.int/health-topics/adolescent-health#tab=tab_1. Compared to developed countries, adolescents living in developing countries generally receive fewer life skills and self-directed education on how to improve lifestyles, and a large number of these adolescents suffer from several health and lifestyle problems such as They suffer from unhealthy food or malnutrition, iron deficiency anemia, inactivity, tooth decay, mood disorders, and poor communication (especially with the opposite sex) and life skills [6–8].

Developmental self-care needs are identified as “conditions related to human developmental processes or conditions and events that occur during various stages of the life cycle.” In adolescents and adults, self-care needs are ideally met through the practice of self-care. When a person’s self-care requirements are met through self-care practice, the goal of self-care is achieved, and when it is not met, there is a lack of self-care [9].

One of the most effective strategies is the use of e-health in the treatment and prevention of health problems in children and adolescents. According to the definition of the World Health Organization (WHO), e-health emphasizes the cost-effective and secure use of information and communication technologies (ICT) in health support, including health services, monitoring, education, knowledge, and related research [10]. Increasing interest in exploiting the capabilities of mobile technology to support health has led to the development of an interdisciplinary branch of mobile health that can support the provision and promotion of health [11]. Mobile health is the provision of health services through mobile phones and their applications. Mobile applications are the ones designed for electronic devices such as smartphones and tablets [12, 13].

Self-care is greeted with more acceptance in the form of health along with providing targeted services, non-stop and interactive. It can increase technical capabilities, that is, the ability to carry it anywhere and anytime and the ability to send information through social networks. It also provides individual feedback and is used as a tool to encourage physical activity and healthy diets as well as monitoring heart disease, diabetes, and asthma symptoms. Furthermore, it can send reminders regarding appointments. Quitting smoking, promoting sexual health, reducing the transmission of sexually transmitted diseases, and assisting with prenatal care are some of the other benefits of self-care [14]. These programs can reduce the cost of health care and promote prevention, management, and treatment of chronic diseases, therefore reducing hospital stays. It also increases the quality of the data gathered, which helps healthcare professionals make faster and more accurate decisions. With the increase in the range of health software, the provision of services by mobile health has developed [15, 16].

A study of a mobile app to promote good oral health behaviors by Scheerman et al. (2018) showed that users welcome and enjoy consulting the app. The “white teeth” program can be included in current orthodontic care. The “white teeth” program includes all the techniques that make it a unique and promising home program to promote oral health among adolescents with fixed orthodontics [17]. A study by Ahmadi et al. (2015) showed that by connecting patients to care providers anywhere and anytime, mobile technologies can quickly access information, reduce costs, facilitate remote care, and increase quality care efficiency [18].

Given the ever-increasing popularity of healthcare software and as part of customer health informatics, these applications have the potential to facilitate patient self-care through patient education, disease management, and tracking. In addition, these apps potentially increase motivational capabilities and improve drug adherence. On the other hand, what is vital for the development of effective self-care as well as health and disease management are supervision of health activities and conditions directly related to the individual’s health goals, understanding and applying areas affecting health activities as well as the latest technologies in the world [16].

The mobile applications available in and out of the country have merely focused on self-care for a specific disease such as diabetes or asthma or for a health problem such as obesity. According to the investigations, a comprehensive mobile application for physical self-care of adolescents was not found. Therefore, the aim of this study was to design a protocol for the design and evaluation of mobile application for adolescents’ physical self-care.

### General goals

This study has three general goals:

1. Design and evaluation of a mobile application for adolescent self-care.
2. Designing and evaluating psychometric properties of “Questionnaire for Measuring Adolescent Self-Care Performance.”
3. Determining the effectiveness of self-care interventions based on mobile application among adolescents.

## Methods

**Fig. 1:**
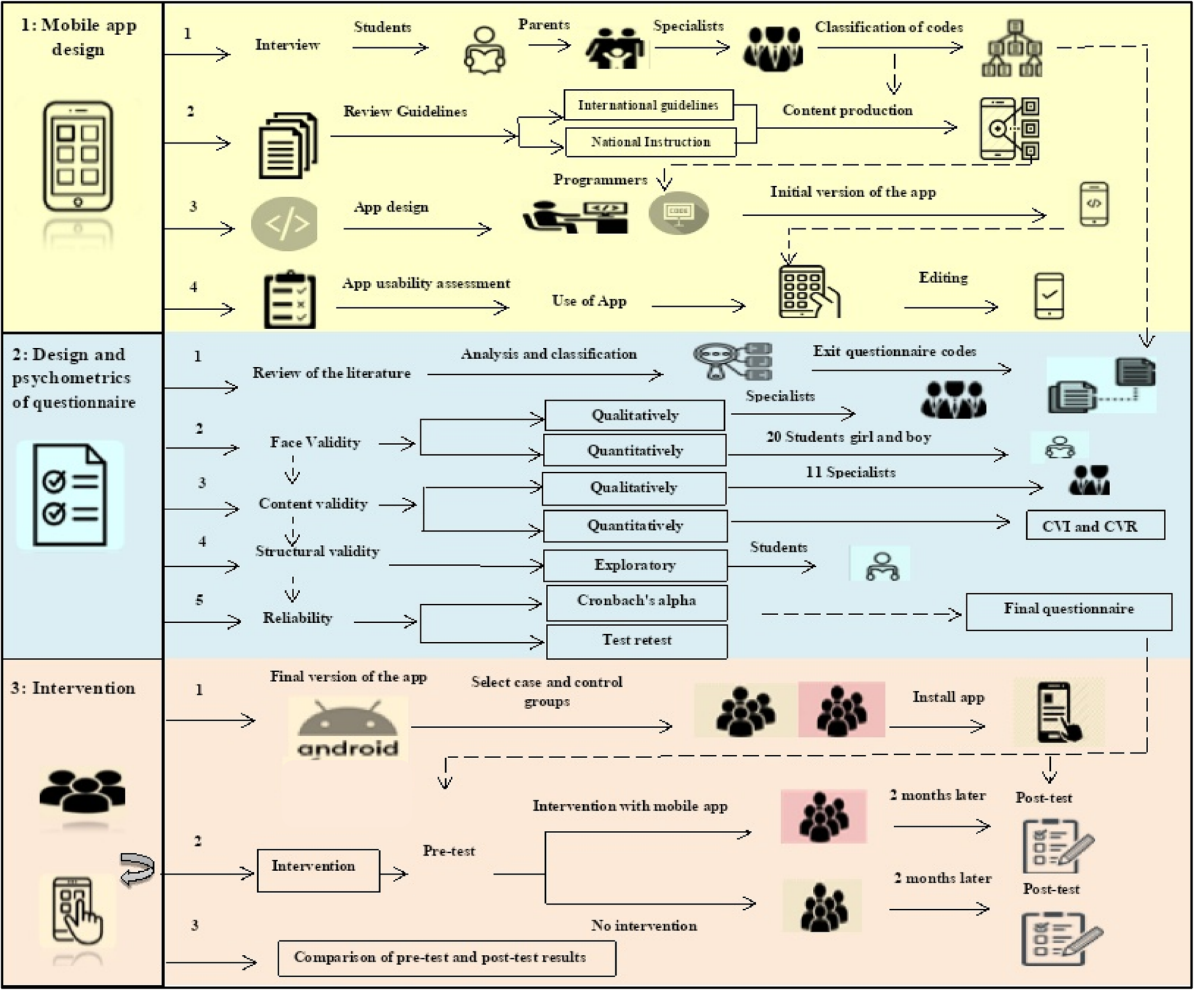
An overview of the study design

The present study is a combination of developmental, applied, methodological, and field trials or studies and will be conducted in three stages. The first stage is to design and evaluate the initial version of a mobile application for adolescent’s self-care. The second stage is the design and psychometrics of a “Questionnaire for Measuring Adolescent Self-Care Performance.” The third stage is determining the effectiveness of the designed application in an interventional study. The target group of this study are adolescents aged 10–19 years old (Fig. 1).

### Inclusion and exclusion criteria

Inclusion criteria are as follows: 10–19 years old, informed consent to participate in the study as well as the consent of their parents or legal guardians, having a smartphone and the ability to work with it. Exclusion criteria are as follows: unwillingness to continue cooperation and incomplete completion of the questionnaire.

### Study population

#### The first stage

In this section, the participants and population under investigation are adolescents (male and female students of Amol City schools), parents (father or mother or both), health professionals (specialists who have experience working in the field of adolescents and programming in the field of health) who are willing to participate and present their experiences and opinions in the field of designing and developing Self-care mobile application.

#### The second stage

The population studied at this stage are adolescents (boys and girls students of Amol schools), parents (father or mother or both), health professionals (specialists who have experience working in the field of adolescents), and the desire to participate and provide experiences and they have their own opinions in the field of questionnaire design.

#### The third stage

The studied population will be all adolescents aged 10–19 years old in Amol City and interested in knowingly participating in the research.

### Sampling method

#### The first stage

The sampling of adolescents is conducted purposefully with maximum diversity (considering factors such as age, sex, educational background, and parents’ education). Also, eligible parents or caregivers of adolescents will be invited and health professionals will be purposefully selected. Then, the interview is carried out until no new code is created through repeated and open questions and the data is saturated. Satisfaction with the study will be obtained before the interview and people will answer general and specific questions about mobile apps during the interview. In the stage of application usability evaluation, 15 male and female users and 15 specialists will be employed.

#### The second stage

To perform construct validity, based on the list prepared by the Department of Education, out of the 337 public schools and 93 non-governmental schools listed, six girls’ schools and six boys’ schools were randomly selected, and from each school and every grade, students of 10–19 years old were selected. The number of samples will be 5–10 times the questionnaire items.

#### The third stage

**Fig. 2:**
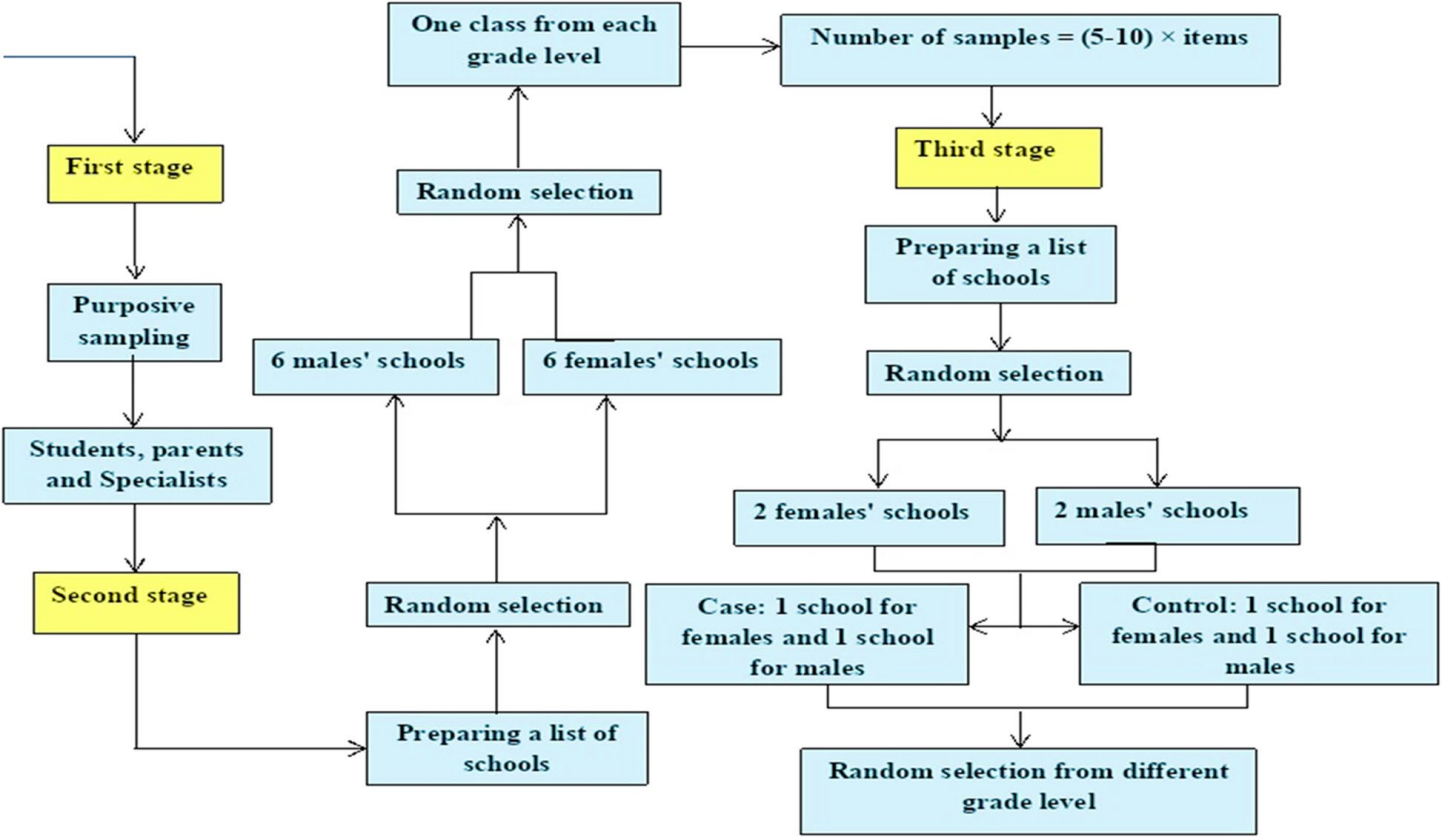
The study sampling stages

The next stage of the sampling method is cluster random. From 337 government schools and 93 non-government schools, based on the list of urban schools received from the Department of Education, two boys’ schools and two girls’ schools are randomly selected. Then, one boy’s school and one girl’s school will be randomly assigned as the intervention group and one boy’s school and one girl’s school will be in the control group, and the sampling of students of different grades of the selected schools will be simple cluster or random. Sampling of students from different grades from selected schools will be simple in cluster or random. The sample size was calculated according to the formula of 43 people in each group. Taking into account 15% attrition, the sample size was determined to be 50 people in each group. The study will be conducted on 100 students of Amol City. Of these, 50 people will be assigned to the intervention group and 50 people will be assigned to the control group. From each school, 25 samples were selected (Fig. 2).

$$n= \frac{{2\left(Z1-\frac{\alpha }{2}+ Z1-\beta \right)}^{2}}{{\left(\frac{\Delta }{S}\right)}^{2}}$$

The schools selected for the intervention and control groups will be far apart in order to prevent contamination bias. The effects of confounding variables such as economic status and social class will be controlled in the clusters in the data analysis with the covariance test. All samples, education office, school managers, and user panel managers will be blinded.

### Study process

#### Stage 1

In the first stage, in line with the principles of user-centered design, the design consists of four key steps:

1. Assess the needs of end users through in-depth interviews with stakeholders
2. Design functional modules and evidence-based content based on the end needs of users
3. Design and development of M-Health system structure and user interface based on the role and characteristics of end users
4. Improving and upgrading the system through the evaluation of the final program

Step 1: This step examines the needs, preferences, and priorities of adolescents, parents, and health professionals about a mobile self-care app for adolescents. At this stage, individual interviews are conducted with parents and adolescents separately. Considering that the purpose of this study is to design a mobile application with the topic of self-care in adolescents, three subgroups of experts, parents, and adolescents can be used for the interview. While the guide questions will have slight differences in the three subgroups, they should cover two sections related to app features and self-care content. In general, the guide questions will be set according to the review of the texts and the expert opinions of the research team (Table 1). For example, in the content section, based on a review of the texts, 5 sections were considered for the physical dimension of self-care, which is discussed in the interviews. During the interviews probing questions can be used to collect more data.

**Table 1:** Interview guide

Adolescents
✔ How do you define self-care?
✔ What measures do you take to take care of yourself? Why?
✔ Who do you think should help you take care of yourself?
✔ What do you think about having a website and smartphone app that can help you better manage your self-care?
✔ How do you want to track information and actions related to self-care in an app? what information?
✔ How do you want to perform better in the app to help you set self-care goals?
✔ If there is to be an app in the field of health with 5 sections (physical activity, nutrition, risky behaviors, and safety and events), what do you think about the educational content of these sections and how to present them?
Parents
✔ How do you define self-care?
✔ How long and how has your adolescent been active in the field of self-care?
✔ Do you think your adolescent wants to develop self-care practices? Why? Why not?
✔ What tools and resources do you feel your adolescent has to manage their own care independently?
✔ What do you think about having a website and smartphone app that can help your adolescent manage and develop better self-care?
✔ How regularly does your adolescent track their self-care activities? (for example, in paper or electronic form) Why not?
✔ About self-care management, what information do you or your adolescent want to feel comfortable with self-care?
✔ If there is to be an app in the field of health with 5 sections (physical activity, nutrition, risky behaviors and safety and accidents), what do you think about the educational content of these sections and how to present them?
Experts
✔ What do you say are the biggest challenges and obstacles for adolescents and families in self-care?
✔ Do you feel they have the tools and resources needed to manage self-care independently?
✔ What do you think about having a website and smartphone app that can help adolescents manage and develop better self-care?
✔ How do you see the main and practical themes of a suitable app for the care of adolescents?
✔ Are there other important features that we haven’t talked about that can help them become healthier?
✔ If there is to be an app in the field of health with 5 sections (physical activity, nutrition, risky behaviors, and safety and accidents), what do you think about the educational content of these sections and how to present them?

Step 2: Content quality is one of the most important aspects of reputable health programs. Interviews and discussions will be held on the main topics of the content and features of the program. To ensure the quality of health information provided in the program, a review of the texts is carried out. Information on the main themes of the self-care program will be obtained from national and international guidelines (WHO, CDC, FDA, ADF, Ministry of Health and Medicine, Health Education, and Health Promotion Office).

Step 3: After specifying the functions and modules, the system architecture is designed. To ensure a user-friendly interface, a smart mobile application will be designed by professional designers and application development experts with full consideration of user needs. The user interface should be designed in a friendly and efficient way, which is critical in improving the user experience in using and interacting with the app. This program has three main innovative features: privacy, program updates, and simple and automatic transfer of self-care programs related to user health status. This app will be designed for the popular Android operating system smartphones. This operating system (OS) has widely been used in mobile phones worldwide, in particular in those which are common in the Iranian market and we chose the Android OS to avoid limitations that could be experienced with other less common OS in our context of use.

Step 4: Evaluate the final program. In this step, the usability of mobile applications for adolescent self-care is evaluated from the perspective of students and health professionals. At this stage, 15 male and female students are randomly selected and 15 health professionals are selected by available sampling and are asked to install the final version of the self-care program on their mobile phones. After 2 weeks of using the program, participants are asked to answer the Mobile Health App Usability Questionnaire (MAUQ). The Mobile Health App Usability Questionnaire (MAUQ) has 3 subscales and 18 questions: the first part is related to ease of use (5 questions), the second part is user interface and satisfaction (7 questions) and the third part is about usefulness (6 questions) [19].

#### Stage II

In the second stage, with an organized review of available sources and articles based on the concept of self-care, the items of the “Questionnaire for Measuring Self-Care Performance in Adolescents” will be extracted. Next, the validity (face, content, and construct) and reliability of the designed questionnaire will be determined.

#### Stage III

The third stage is an interventional method of experimental study. This research will be conducted through a pre-test-post-test design with a control group. All the students studying in schools constitute the research community. The researcher first introduces the adolescents to the full explanation of the research and its goals. Then informed written consent will be obtained from the volunteers. None of the participants will be subject to any other research intervention. Schools will be randomly assigned to one of the two intervention (group 1) and control (group 2) groups. The full content of the program will be visible only to the participants in the intervention group. Participants in the control group will be able to download the app after the end of the intervention to use the educational benefits of the app. A self-care application can be used online and offline designed for smartphones running the Android operating system. To run this test, eligible students are asked to install the self-care app on their smartphone and see the necessary instructions on how to work with the program in the form of a short guide on the main page of the application. Initially, the participants are asked to complete the self-care questionnaire in the form of a pre-test. Then, the educational content designed for the application is provided for individuals based on national and international guidelines and protocols of (WHO, CDC, FDA, ADF, Ministry of Health and Medicine, Health Education and Health Promotion Office). This program will be available to people for 3 months and after installing the application on their smartphone and entering the information requested from the users, educational content and reminder messages will be sent according to their conditions. In the reminders section, the user registers his health behavior based on the day, time, and number of times he wants, and a reminder is sent to him. But if the user’s self-care status after answering the self-care questions in each of the 5 dimensions of self-care is assessed as weak or average, motivational health messages are sent to the user daily in the form of notifications, so that the user is encouraged to perform self-care behaviors. The use of the application by the participants is reviewed in the management panel and health messages and incentives are provided for the use of this program. To provide support, the researcher’s phone number is given to the subjects to make a call in case of problems. Three months after the intervention, when no new content is sent to the student, the participants in both groups are requested to complete the self-care questionnaire in the form of a post-test. After the end of the data collection process, the individuals in the control group can install a mobile Application and the necessary training will be given to them after the end of the study. In the present study, content, information, and experiences reach the end users or audience with the specific purpose of self-care through mobile applications in the form of text, audio, or other art forms. Users find and use health-related self-care information through content. The graphic design of the application is based on the opinion of the research team professional designers and application development experts. The production of the content is based on the points extracted from the interviews, national and international guidelines, and protocols of (WHO, CDC, FDA, Ministry of Health and Medicine, Health Education and Health Promotion Office), for example, Australian dietary guidelines, Canberra: NHMRC [20], WHO guidelines on physical activity and sedentary behavior for children and adolescents aged 5–17 years: summary of the evidence [21], and physical activity guidelines [22].

### Data collection methods

#### The first stage

At this stage, data is collected separately through in-depth semi-structured interviews with adolescents, parents, and health professionals. Informed consent will be obtained before the interview. Interviews will be conducted by the researcher, using the interview guide questions. To minimize the differences between the interviewer and the respondents, the interviewer will make use of ice-breaking questions, appropriate language, active listening, and calm body language. All interviews will be transcribed verbatim. They will be analyzed and the codes will be extracted.

#### The second stage

At this stage, to determine construct validity, based on the list prepared by the Department of Education, six girls’ schools and six boys’ schools were randomly selected. From each school and each grade—within the 10–19-year-old age group—a class will be selected in a cluster random sample. The number of samples per item will be 5–10 samples. At this stage, we collect data using a questionnaire prepared in the previous stage, which has both face and content validity.

#### The third stage

At this stage, we use a researcher-made questionnaire the validity and reliability of which were determined in the second stage. The desired questionnaire is uploaded on the application and after installation, this program will be available to people for 3 months. After 3 months of the intervention, by an SMS or a phone call, the people of both groups are asked to complete the questionnaire uploaded, which is in the form of a post-test, and send it back to the researcher. After collecting the data, analysis is conducted by statistical tests.

### Methods of analysis

In the first stage, after the interviews are completed, all the interviews will be transcribed verbatim and entered into NVIVO 12 (Qualitative Analysis Software) for data management. Demographic data will be encoded and analyzed using Microsoft Excel software to determine central indicators and values in a sampling distribution. The data will be analyzed to provide a simple descriptive summary of participants’ opinions presented in everyday language. Specifically, the data will be coded for all the participants and classified to reflect the main themes. In the second stage, we employ factor analysis which is a statistical method that determines the number and nature of variables that a test measures. Following this method, variables with homogeneous correlations are categorized in the form of new variables called factors. The purpose of exploratory factor analysis is to discover the dimensions and common applications including instrument dimensions, standardization of questionnaires or tests, dimensionality, and evaluation of consistency and differentiation in the discussion of construct validity [23]. In the third stage, descriptive statistics (frequency, frequency percentage, mean, and standard deviation) will be used to analyze the data. The Kolmogorov–Smirnov test will also be used to check the normality of quantitative variables. Between-group and within-group comparisons about quantitative variables, independent *t*-test and paired *t*-test, and analysis of variance will be calculated. The chi-square test and Fisher’s exact test in SPSS 16 software will be utilized to test the homogeneity of qualitative variables between the two groups.

### Ethical considerations

- ➣ All aspects of human subject research ethics will be fully observed in this research. All exemptions and approvals will be checked and observed by the researchers. Also, this research has a code of ethics from Shahid Beheshti University of Medical Sciences. IR.SBMU.PHNS.REC.1400.073(.
- ➣ Informed consent will be obtained from all study participants at all stages. In addition, informed consent will be obtained from adolescents, their parents, or legal guardians to participate in the study. Also, in the initial informed consent received from the participants, it will be mentioned that the possibility of secondary analysis is possible without additional consent. Participants will also be assured of their voluntary participation in the research and their freedom to withdraw from the study at any stage of the research.
- ➣ The data obtained from the study remains confidential and anonymous in the study. Participants will also be assured that the information will remain confidential. In the reports and presentation of the results, no connection between the data and the profile of the participant will be given, so that the participants will not be identified.
- ➣ This study will not involve any therapeutic or invasive procedures. During the implementation of the research, there will be no threat of physical, financial, or social harm to people.

## Results

Our findings are scheduled for a full analysis, with expectations that analyses will be completed by September 2023. We intend to publish results in peer-reviewed journals.

It is expected based on the objectives of the study:

The self-care mobile application should be designed in a scientific and user-oriented way and should be usable in the target group. We also expect users to be satisfied with the application, consider it useful, and evaluate it as easy to use.

Also, we expect that at the end of the research, a questionnaire measuring the self-care performance of adolescents will be designed, and it will have appropriate validity and reliability, and it can be used in other studies and populations.

Based on the objectives of the third stage of the study, we expect that the educational intervention in the intervention group, through the use of the designed mobile application, will improve self-care behaviors in terms of physical activity, nutrition, personal hygiene, risky behaviors, and safety and events.

## Discussion

In the first stage, based on the opinions received from the target group, a user-centered educational application for the self-care of adolescents will be designed. In the second stage, after determining the validity and reliability, a questionnaire will be designed to measure the self-care performance of adolescents. In the third stage, using an intervention study for 3 months, the effectiveness of the training will be determined through the designed application.

In the context of the goal of the first stage, our goal in the first stage is to design an application for the self-care of adolescents so that this application is comprehensive and practical and based on the needs and tastes of users. Therefore, it should be noted that designing a mobile phone application by involving users is one of the points that should be considered in the design of health applications. Mobile health projects (mHealth) apply user-centered design (UCD) using patient inputs, careers, physicians, and other stakeholders throughout the project life cycle to create better designs that ultimately could improve efficiency and effectiveness [24–28]. Williams et al. (2014) emphasized that mobile health-tracking applications play a supportive role in self-management rather than replacing current care. Therefore, user-centered mobile health tracking applications may be effective not as stand-alone treatments, but as adjunctive treatments [29]. The study by Young Kim et al. (2020) showed that there were barriers such as security issues, application costs, and the need for user-friendly designs and reliable information for optimal patient use. In addition, healthcare professionals should consider the needs and preferences of patients to promote the acceptance of mobile applications [30]. The goals and results of these two studies are in line with the aim of the 1 stage of our research. In the context of the goal of the second stage, questionnaires are the main means of data collection and there is an increasing need for different questionnaires. Maneesriwongul believes that in the process of questionnaire making, cultural background differences should be taken into account along with the translation of the questionnaire, and the questionnaire should be compatible with the culture of the target community to be valid and reliable. In addition, an assessment questionnaire should be relevant and easily applicable to the target population [31, 32]. In the context of the goal of the second stage, the “Child and Adolescent Self-Care Performance Questionnaire” (CASPQ) examines self-care performance at the ages of 9–18 years in 3 dimensions (global self-care needs, developmental self-care needs, and health deviation self-care needs) using 35 items [33]. Fernández et al. investigated the validity and reliability of this questionnaire in 489 children aged 8–12 years. The results showed that CASPQ shows sufficient metric properties similar to the original questionnaire. For this reason, it is a useful questionnaire for evaluating self-care practices and planning interventions aimed at improving them [34]. The purpose and results of this study are consistent with the purpose of the current study, which is to design a questionnaire to measure self-care performance, but this questionnaire is not comprehensive. Meanwhile, the Congenital Heart Disease Self-Care Questionnaire (CHDSCQ), includes 45 items with 4 subscales (knowledge, adherence, symptom recognition, and health maintenance behaviors), and evaluates self-care status in adolescents and young adults with congenital heart disease. The questionnaire takes approximately 15 min to complete, and higher scores indicate better self-care. In a psychometric study, Pike et al. (2019) examined the self-care questionnaire of adolescents and young adults with congenital heart disease. The results showed that the CHDSCQ is the first valid questionnaire (both content and criteria/clinical validation) to assess self-care in the CHD population [35]. This questionnaire is designed for adolescents with heart disease, which only examines the self-care status related to heart disease in patients and does not have the necessary capacity to evaluate self-care in healthy adolescents. The purpose and results of this study are contrary to the purpose of the current study, which is to design a questionnaire to measure self-care performance in healthy adolescents. In the context of the goal of the third stage, Thornton et al. (2021) conducted a study aimed at multiple health behavior change, a self-monitoring mobile app for adolescents: A development and usability study of the Health4Life app. The Health4Life program is a smartly designed, self-monitoring program for adolescents that simultaneously targets the Big 6 lifestyle behaviors. Adolescents evaluated this program as very acceptable and usable. This program has the potential to effectively modify important risk factors for chronic disease among youth [36]. Also, a study by Alves et al. (2021) was conducted to develop and validate health technology to promote self-care for adolescents with diabetes. The results showed that the use of this program by adolescents, considering that it is a very understandable electronic technology, helps to acquire new knowledge and adhere to healthy practices [37]. The purpose and results of these two studies are consistent with the purpose of the present study, in the field of using a health application to promote health in adolescents.

Also, the study by Antunes et al. (2023) aims to evaluate the impact of an educational intervention associated with physical exercise based on the web in promoting health and quality of life of patients with fibromyalgia in Brazil. which is consistent with the current research in terms of Online educational intervention for self-care and health promotion [38].

From the limitations of this research, it can be said that because little research has been done in the field of application design to improve the health of adolescents, the possibility of comparing the results will be limited. Considering that adolescents are active users of mobile phone applications, it is recommended to design more applications in the field of adolescent health. One of the strengths of this research study is the novelty of the method used to institutionalize self-care and assess adolescents’ health by contemporary technologies, namely mobile applications. Application design with a user-centered approach (in this study, parents, students, and professionals) is another strength of this research. In addition, this research provides a suitable platform for various interventions for adolescents and schools. This method can also be used for other age groups or other diseases and health problems.

This study is an integration of mobile technologies, health education, and health promotion programs in the form of mobile health. Given that the number of mobile phone users is increasing day after day and so is their popularity, it seems that using applications in the field of health seems to be the right decision. The researchers also tried to keep in mind the following considerations such as the application design process, criteria such as user participation, content quality, usability, the need for the program to match the literacy level of users, program security, user privacy, and the possibility of updating the content without the need to update the entire program. The findings of this study can be useful in designing and formulating policies, applications, and programs in the field of health and on how to monitor and guide the health of individuals.

### Trial status

Protocol version number: May 12, 2023

First day of recruitment: July 26, 2022

Expected end of the intervention: December 31, 2023

#### Implications

Among the possible implications, we can point out some such as using the application at the level of the Ministry of Health, and Education, as well as research centers and health professionals to promote adolescent health. This study helps the society to improve the level of self-care behaviors of adolescents and as a result, by reducing chronic diseases, they are healthier in adulthood and have a healthier society. Also, experts can use the results of research related, to discover the needs and gaps in the health of adolescents and develop more appropriate educational plans. On the other hand, the results can help the health system in the direction of health policies, reducing the disease and financial burden.

## Data Availability

Following analysis, an anonymized data set will be made available to interested fellow investigators by the corresponding author upon reasonable request.

## Abbreviations

WHO: World Health Organization
ICT: Information and communication technologies
CASPQ: Child and Adolescent Self-Care Performance Questionnaire
CDC: Centers for Disease Control and Prevention
FDA: Food and Drug Administration
NHMRC: National Health and Medical Research Council
UCD: User-centered design
CASPQ: Child and Adolescent Self-Care Performance Questionnaire
CHDSCQ: Congenital Heart Disease Self-Care Questionnaire
